# [Retracted] MicroRNA-490-3p regulates cell proliferation and apoptosis in gastric cancer via direct targeting of AKT1

**DOI:** 10.3892/etm.2025.13010

**Published:** 2025-11-04

**Authors:** Hong Yu, Jianying Sun, Shaochang Jiang, Ying Xu

Exp Ther Med 17:1330–1336, 2019; DOI: 10.3892/etm.2018.7042

Following the publication of this paper, it was drawn to the Editor’s attention by a concerned reader that, for the colony formation assay data shown in Fig. 2C, the ‘inhibitor-NC’ and ‘mi-inhibitor’ images looked unexpectedly similar, considering that these were intended to have shown the results from differently performed experiments. Upon performing an independent analysis of the data in this paper in the Editorial Office, it was subsequently revealed that some of these same data, in addition to western blot data in Fig. 3E, had already been submitted for publication in articles written by different authors at different research institutes.

In view of the fact that the abovementioned data had already apparently been published previously, the Editor of *Experimental and Therapeutic Medicine* has decided that this paper should be retracted from the Journal. The authors were asked for an explanation to account for these concerns, but the Editorial Office did not receive a reply. The Editor apologizes to the readership for any inconvenience caused.

